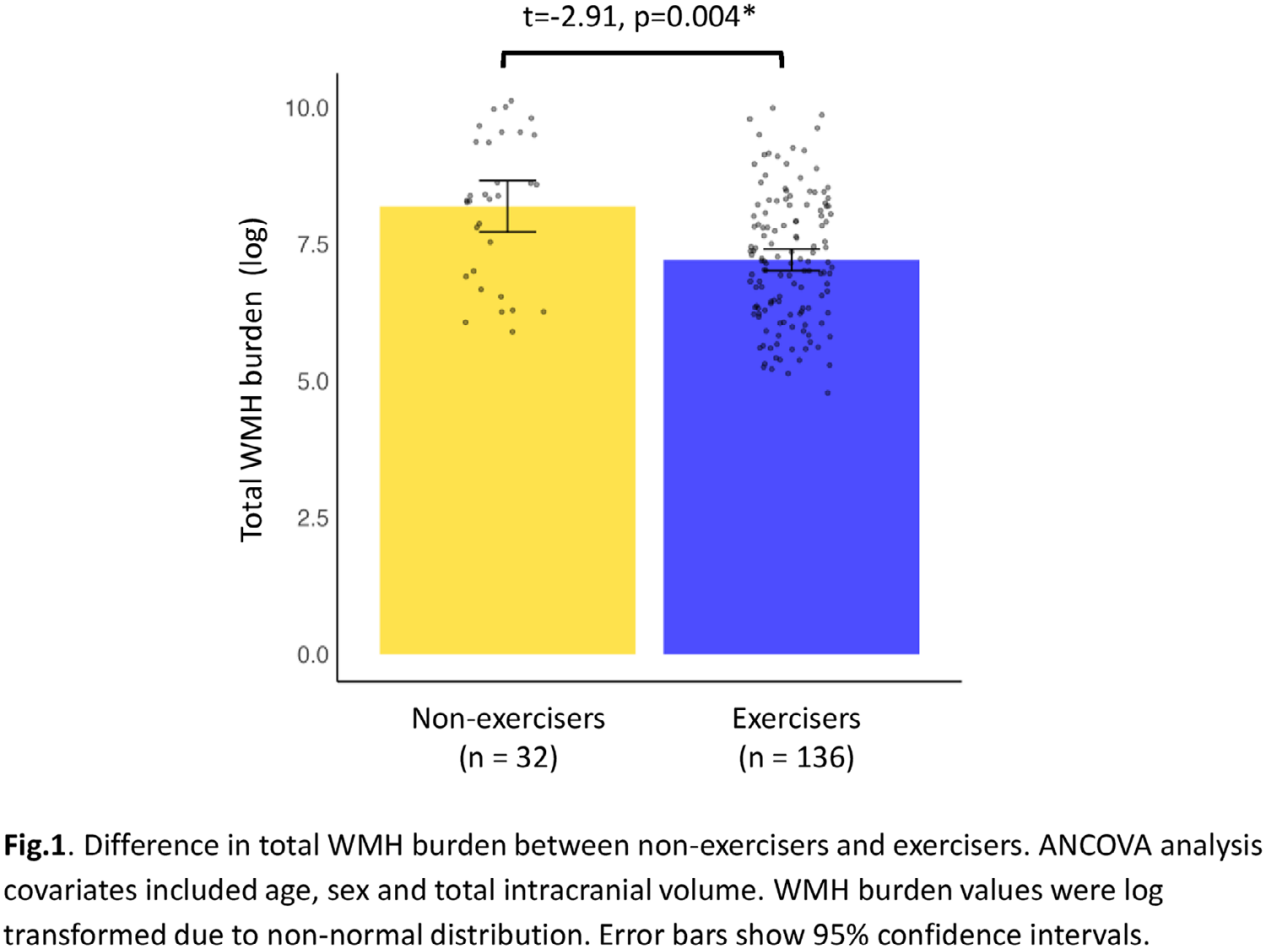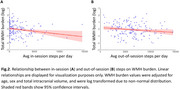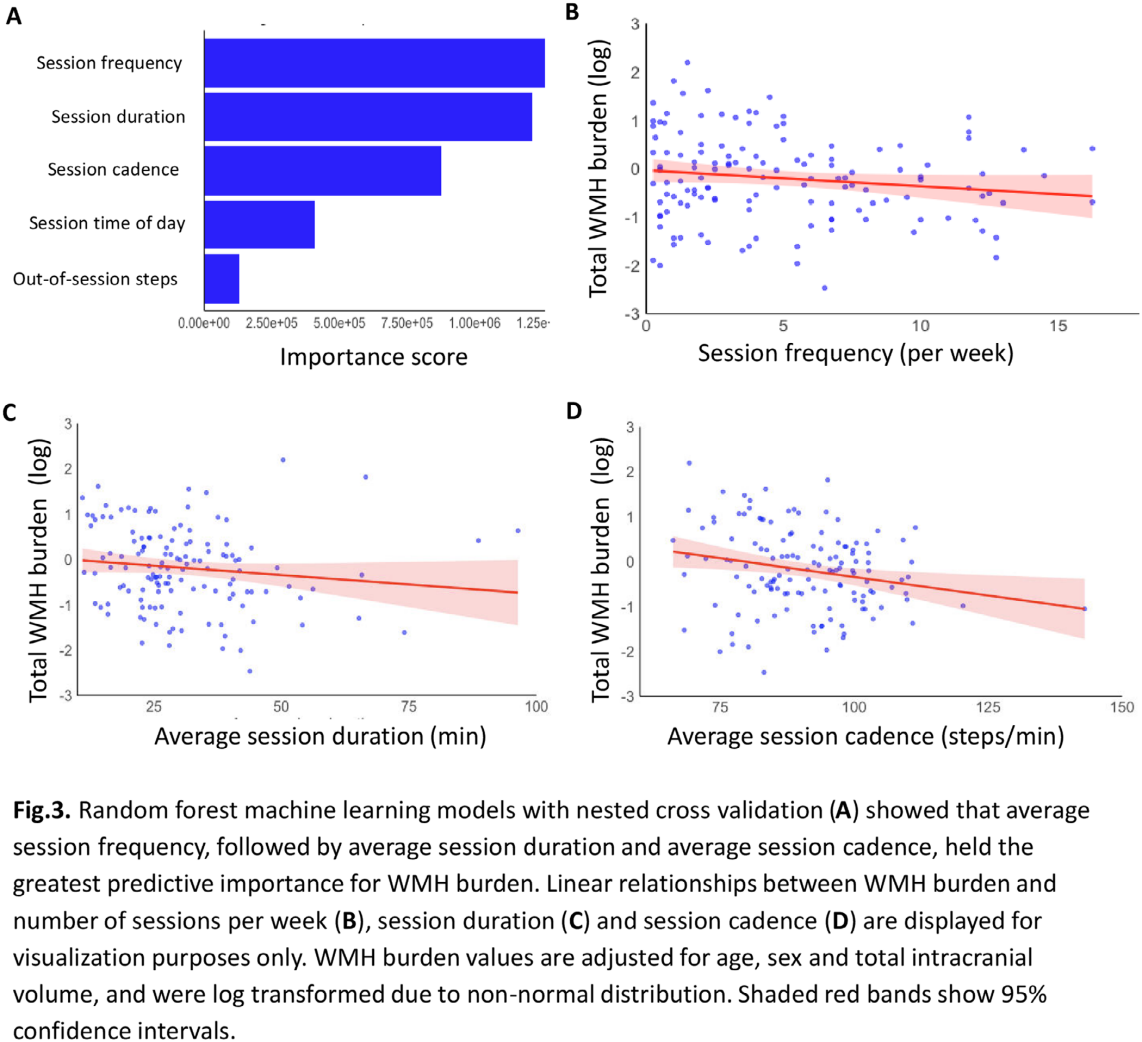# Physical activity session parameters predict white matter hyperintensity burden in older adults living without dementia

**DOI:** 10.1002/alz70860_106831

**Published:** 2025-12-23

**Authors:** Claire J. Cadwallader, Anna M. VandeBunte, Rowan Saloner, Coty Chen, Valentina E. Diaz, Molly Olzinski, Gil D. Rabinovici, Joel H Kramer, Kaitlin B Casaletto, Emily W. Paolillo

**Affiliations:** ^1^ Memory and Aging Center, UCSF Weill Institute for Neurosciences, University of California, San Francisco, San Francisco, CA, USA; ^2^ Memory and Aging Center, Weill Institute for Neurosciences, University of California San Francisco, San Francisco, CA, USA; ^3^ Global Brain Health Institute, University of California, San Francisco, San Francisco, CA, USA; ^4^ UCSF Alzheimer's Disease Research Center, San Francisco, CA, USA; ^5^ Global Brain Health Institute, University of California San Francisco, San Francisco, CA, USA

## Abstract

**Background:**

Higher levels of physical activity (PA) are associated with better cognition and brain health in older adults. However, the importance and optimal parameters (e.g. intensity, duration, frequency) of structured PA sessions remain unclear. We isolated PA sessions from actigraphy data in a group of older adults to 1) compare cognitive, brain volume and plasma biomarker outcomes between participants who engaged in sessions (exercisers) and those who did not (non‐exercisers), 2) examine the relative importance of in‐session vs. out‐of‐session activity on outcomes, and 3) in exercisers, examine the relative importance of specific session parameters on outcomes.

**Method:**

329 older adults without dementia (mean age=71.3±11.6; 57% female; 14% CDR=0.5) completed 30 days of actigraphy monitoring, blood draws with plasma biomarker assays (*p*‐tau181, GFAP, NfL, AB40, AB42), brain MRI, and neuropsychological evaluation. PA sessions were defined as ≥10 consecutive minutes where step count >40 steps/min, previously established as a threshold for purposeful/low intensity PA. Session parameters included average total in‐session steps/day, total out‐of‐session steps/day, session duration (mins), cadence (steps/min), frequency (sessions/week), and time of day (AM/PM). ANCOVA compared exercisers vs. non‐exercisers on brain health outcomes including memory, executive function, frontal and medial temporal lobe volumes, total white matter hyperintensity (WMH) burden and plasma biomarkers. Random forest machine learning with nested cross‐validation examined the importance of in‐session vs. out‐of‐session steps in the full sample, and specific session parameters in exercisers only.

**Result:**

Exercisers showed lower WMH burden than non‐exercisers when controlling for age, sex, and total intracranial volume (t=‐2.91, *p* = 0.004; Figure 1). In‐session steps were more predictive of WMH burden than out‐of‐session steps (Figure 2A, B). In models examining session parameters in exercisers (Figure 3A), number of sessions per week (Figure 3B) followed by session duration (Figure 3C) and cadence (Figure 4D) were the most important predictors of WMH burden. Models for other outcomes showed poor predictive power.

**Conclusion:**

PA during structured sessions appeared most important for WMH burden in older adults, a key marker of brain aging and cerebrovascular health. Frequency of session engagement may be the most critical session parameter for PA optimization. Future work will seek to optimize parameter cut‐offs to inform more precise interventions and recommendations for brain health.